# Microwave-assisted biosynthesis of silver nanoparticles using two marine microalgal extracts and their antimycobacteriosis activity against bacteria isolated from *Betta splendens*

**DOI:** 10.1038/s41598-025-00128-w

**Published:** 2025-05-01

**Authors:** Piyapan Manklinniam, Saranya Phunpruch, Aparporn Sakulkalavek, Rachsak Sakdanuphab, Worakrit Worananthakij

**Affiliations:** 1 Department of Biology, School of Science, King Mongkut’s Institute of Technology Ladkrabang, Bangkok, 10520 Thailand; 2 Bioenergy Research Unit, School of Science, King Mongkut’s Institute of Technology Ladkrabang, Bangkok, 10520 Thailand; 3 Department of Physics, School of Science, King Mongkut’s Institute of Technology Ladkrabang, Bangkok, 10520 Thailand; 4 Electronic and Optoelectronic Device Research Unit, School of Science, King Mongkut’s Institute of Technology Ladkrabang, Bangkok, 10520 Thailand; 5 School of Integrated Innovative Technology, King Mongkut’s Institute of Technology Ladkrabang, Bangkok, 10520 Thailand

**Keywords:** Biosynthesis, Silver nanoparticles, Microwave radiation, Mycobacteriosis, Antibacterial activity, Biotechnology, Microbiology

## Abstract

This study explores the synthesis and antibacterial properties of silver nanoparticles (AgNPs) as a safer, eco-friendly alternative to traditional chemical treatments for bacterial infections. AgNPs were synthesized using aqueous extracts of marine microalgae, *Isochrysis galbana* and *Chaetoceros calcitrans*, via conventional and microwave-assisted methods, with the latter accelerating nanoparticle production. Extracts in ethanol, hexane, and acetone were tested, with the ethanolic extract of *I. galbana* showing the strongest antibacterial effects. The AgNPs exhibited broad-spectrum antibacterial activity against pathogens such as *Staphylococcus aureus*, *Bacillus subtilis*, *Escherichia coli*, *Pseudomonas aeruginosa*, and fish pathogens like *Aeromonas veronii*. Microwave-assisted synthesis with ethanolic extracts resulted in the highest inhibition, particularly against fish and tuberculosis-related pathogens, including *Mycobacterium marinum*. Nanoparticle formation was confirmed using various characterization methods, including ultraviolet-visible (UV-Vis) spectroscopy, X-ray diffraction (XRD), fourier transform infrared spectroscopy (FTIR), scanning electron microscopy (SEM), which revealed crystalline structures. Transmission electron microscopy (TEM) analysis revealed that AgNPs varied in size, with an average diameter of less than 50 nm and all particles being smaller than 100 nm. This research demonstrates the potential of AgNPs as an effective alternative to antibiotics, offering targeted bacterial inhibition while reducing the risk of antibiotic resistance. This makes it a promising approach for treating bacterial infections in ornamental fish.

**Supplementary Information:**

The online version contains supplementary material available at 10.1038/s41598-025-00128-w.

## Introduction

Mycobacteria are a group of Gram-positive bacteria widely distributed in the environment and are known to cause a variety of infectious diseases in both animals and humans. In the aquaculture industry, mycobacteria are recognized as significant causative agents of mycobacteriosis in various fish species, including ornamental fish^1–4^. As a result, mycobacteriosis in aquatic animals negatively impacts product prices. Moreover, granulomatous disease in fish is zoonotic, posing a substantial risk to humans and necessitating proper treatment^5^. However, mycobacteriosis in fish has not been effectively treated with antibiotics. Consequently, both natural and synthetic substances are being investigated as alternative treatments for mycobacteriosis.

Potential substances for controlling mycobacterial strains include plant extracts, yeast extracts, and nanoparticles synthesized from plants and microbes^6^. Among these, nanoparticle-based treatments offer several advantages, such as prolonged action time and flexibility in administration through various routes^7^. Silver nanoparticles (AgNPs) have been shown to be highly effective antimicrobial agents, even at low concentrations, due to their ability to inhibit the growth of antibiotic-resistant bacteria^8^. AgNPs can penetrate bacterial cells because of their small size, disrupting the cell wall. Smaller nanoparticles are more toxic to bacteria than larger ones^9^, yet they exhibit relatively low toxicity to mammals^10,11^.

One method of synthesizing AgNPs involves the use of bioactive compounds from extracts containing stabilizing and capping agents^12^. Biosynthesized AgNPs exhibit multiple functions, including antiparasitic, antifouling, and antimicrobial activities against bacteria such as *Staphylococcus aureus*,* Escherichia coli*,* Pseudomonas aeruginosa*, and *Bacillus rhizoids*^13^, as well as fungi such as *Aspergillus* sp. and *Rhizopus* sp.^14^. AgNPs synthesized via biosynthesis demonstrate significant antibacterial properties, enhanced by the presence of diverse bioactive compounds on their surfaces^15^. Previous studies have shown that algal extracts can reduce metal ions, leading to the production of metallic nanoparticles^15,16^. Some microalgae are capable of absorbing metal ions and producing metal nanoparticles as part of their detoxification processes^8^. Marine microalgae, such as *Chlorella*, *Isochrysis*, *Chaetoceros*, *Nannochloropsis*, and *Skeletonema*, contain various biochemical compounds with antioxidant and antibacterial properties^17^. Nanoparticles synthesized from algae have demonstrated potential efficacy against microorganisms^18^.

Among the most popular ornamental fish species, Siamese fighting fish, or betta fish, hold high commercial value in many countries. However, it is susceptible to mycobacteriosis, which diminishes its market value. To date, few studies have explored the use of metal nanoparticles synthesized by algae to combat mycobacteriosis. This research aims to: (1) biosynthesize AgNPs using microalgal extracts obtained from *Isochrysis galbana* and *Chaetoceros calcitrans* with ethanol, hexane, and acetone, employing both conventional method and microwave irradiation; (2) characterize the biosynthesized AgNPs using UV-Vis spectroscopy, XRD, FTIR, SEM, and TEM to study their structural changes, stability, and physicochemical properties; and (3) evaluate the antibacterial properties of the biosynthesized AgNPs against common pathogenic bacteria, including *E. coli*, *P. aeruginosa*, *S. aureus*, and *B. subtilis*, as well as bacteria isolated from Siamese fighting fish, such as *A. veronii* and *M. marinum.*

## Materials and methods

### Ethical considerations

This study on the bacterial isolation method from Betta fish was approved by the Research Ethics Committee and the Animal Care and Use Committee at King Mongkut’s Institute of Technology Ladkrabang, Bangkok, Thailand. The reference number on the Certificate of Approval is ACUC-KMITL-RES/2020/008. The ARRIVE guidelines were followed in reporting this work, and all methods were performed in accordance with the relevant guidelines and regulations.

### Algal strains and cultivation

The marine microalgae *I. galbana* and *C. calcitrans* were sourced from the Institute of Marine Science at Burapha University, Chonburi Province, Thailand. The algae were cultured in a 6 L plastic aquarium containing 4 L of Guillard’s F/2 medium^19^. The cultivation was conducted at room temperature under white fluorescent light with an intensity of 2,400 lx, following a light cycle of 18:6 h for a duration of 10 days.

### Preparation of algae aqueous extracts

*I. galbana* and *C. calcitrans* were cultured in Guillard’s F/2 medium under the previously described conditions for 10 days. Cells were harvested by centrifugation (Hermle Z513K) at 2,000 × g at 4 °C for 15 min. The cell pellets were dried at 45 °C for 5 h and then ground using a mortar and pestle. Five grams of the microalgal powder was mixed with 50 mL of different solvents, 95% (v/v) ethanol, hexane, and acetone (Qchemical Co., Ltd.) and incubated at 25 ± 5 °C for 24 h. The ethanolic extracts of *I. galbana* and *C. calcitrans* were designated IsoEt and ChaEt, respectively. The hexane extracts were abbreviated as IsoHe and ChaHe, whereas the acetone extracts were labeled IsoAc and ChaAc, respectively. The resulting extracts were filtered through Whatman No. 1 filter paper and stored in darkness at 4 °C until further use.

### Biosynthesis of AgNPs via algal extracts

AgNPs were synthesized from marine microalgal extracts using two methods: with and without microwave irradiation. For the conventional method (without microwave irradiation), 10 mL of algal extract was added to 90 mL of either 1 mM or 10 mM AgNO_3_ solution, abbreviated as 1 or 10, respectively. The mixture was incubated in darkness at 25 ± 5 °C for 24 h. For AgNPs synthesis using microwave irradiation, the mixture underwent five cycles of microwave exposure, each consisting of 30 s on, and 50 s off. A control experiment was conducted by mixing AgNO_3_ solution (1 mM or 10 mM) with 0.1 M NaOH using a magnetic stirrer at a ratio of 1:4. This mixture was heated to 75 °C for 1 h and then allowed to cool, followed by stirring for an additional 4 h. A visual color change in the solution indicated the formation of AgNPs. AgNPs were collected by centrifugation at 2,000 × g for 30 min, washed twice with distilled water, and dried at 45 °C for 1 h. The abbreviations of AgNPs synthesized by different methods are summarized in Table 1.

**Table 1:** Abbreviations used for samples in this study.

Sample	Aqueous extract		AgNO_3_ (mM)	Method
	Algae	Solvent		
1IsoEt	*I. galbana*	Ethanol	1	synthesis via conventional method
M1 IsoEt	*I. galbana*	Ethanol	1	synthesis via microwave irradiation
10IsoEt	*I. galbana*	Ethanol	10	synthesis via conventional method
M10 IsoEt	*I. galbana*	Ethanol	10	synthesis via microwave irradiation
1IsoHe	*I. galbana*	Hexane	1	synthesis via conventional method
M1 IsoHe	*I. galbana*	Hexane	1	synthesis via microwave irradiation
10IsoHe	*I. galbana*	Hexane	10	synthesis via conventional method
M10 IsoHe	*I. galbana*	Hexane	10	synthesis via microwave irradiation
1IsoAc	*I. galbana*	Acetone	1	synthesis via conventional method
M1 IsoAc	*I. galbana*	Acetone	1	synthesis via microwave irradiation
10IsoAc	*I. galbana*	Acetone	10	synthesis via conventional method
M10 IsoAc	*I. galbana*	Acetone	10	synthesis via microwave irradiation
1ChaEt	*C. calcitrans*	Ethanol	1	synthesis via conventional method
M1 ChaEt	*C. calcitrans*	Ethanol	1	synthesis via microwave irradiation
10ChaEt	*C. calcitrans*	Ethanol	10	synthesis via conventional method
M10 ChaEt	*C. calcitrans*	Ethanol	10	synthesis via microwave irradiation
1ChaHe	*C. calcitrans*	Hexane	1	synthesis via conventional method
M1 ChaHe	*C. calcitrans*	Hexane	1	synthesis via microwave irradiation
10ChaHe	*C. calcitrans*	Hexane	10	synthesis via conventional method
M10 ChaHe	*C. calcitrans*	Hexane	10	synthesis via microwave irradiation
1ChaAc	*C. calcitrans*	Acetone	1	synthesis via conventional method
M1 ChaAc	*C. calcitrans*	Acetone	1	synthesis via microwave irradiation
10ChaAc	*C. calcitrans*	Acetone	10	synthesis via conventional method
M10 ChaAc	*C. calcitrans*	Acetone	10	synthesis via microwave irradiation
1 mM AgNPs	-	-	1	synthesis via conventional method
10 mM AgNPs	-	-	10	synthesis via conventional method

### Characterization of AgNPs

The reduction of Ag⁺ ions in solution was monitored via a UV-Vis spectrophotometer (Shimadzu, UV-1601, Japan). The absorption spectra of AgNPs solution were measured over a wavelength range of 300–750 nm. The dried AgNPs powders were then used for further characterization. The morphology and size of AgNPs were analyzed by SEM (FEI, Quanta 250, USA). TEM was used to determine size, shape, and morphology of the synthesized AgNPs. Samples were analyzed using an FEI TECNAI G2 20 transmission electron microscope, operating at 200 kV. Prior to TEM analysis, a small amount of the synthesized AgNPs was dispersed onto a 200-mesh copper TEM grid and coated with a thin layer of lacy carbon to enhance sample stability and imaging quality. TEM images were analyzed using ImageJ software to manually measure and estimate particle sizes. The dried biomass, following reduction, was subjected to FTIR to identify the chemical bonds and functional groups associated with the AgNPs. FTIR analysis was performed in the range of 400–4,000 cm^–1^ at a resolution of 4 cm^–1^ using an FTIR spectrometer (Nicolet 6700, Thermo Scientific). XRD patterns of the synthesized particles were obtained using a Rigaku SmartLab^®^ X-ray diffractometer. The instrument employed Cu Kα radiation in a θ-2θ configuration and was operated at 40 kV and 30 mA. XRD analysis was used to confirm the crystalline structure of AgNPs.

### Antibacterial activity test

#### Preparation of bacterial inoculum

Two pathogenic bacteria isolated from betta fish, *Aeromonas veronii* WWKMITL-02 (NCBI accession number LC853089) and *Mycobacterium marinum* WWKMITL-03 (NCBI accession number LC853090), were used in this study. These isolates were provided by the Laboratory of Fish Diseases, Department of Biology, King Mongkut’s Institute of Technology Ladkrabang, Bangkok, Thailand. These fish pathogens were selected due to their significant impact on aquaculture, particularly in ornamental fish farming. *A. veronii*^20^ is noteworthy for its pathogenicity, while *M. marinum*^4^ is frequently isolated from diseased ornamental fish and is associated with virulence factors and antimicrobial resistance.

*A. veronii* WWKMITL-02 was cultured on Tryptic Soy Agar (TSA) at 30 °C overnight, while *M. marinum* WWKMITL-03 was grown on Ogawa egg medium^21^ at 30 °C for 3 weeks. Additionally, four test pathogens, *Staphylococcus aureus* TISTR 746, *Bacillus subtilis* TISTR 1248, *Escherichia coli* TISTR 074, and *Pseudomonas aeruginosa* TISTR 2370, were cultivated on TSA at 37 °C for 18 h^20^. These pathogenic strains were chosen due to their frequent use as model organisms in antibacterial studies and their clinical relevance^9,11^. All bacterial strains were cultured for 18 h, except for *M. marinum*, which required 3 weeks of incubation prior to further testing. For the direct suspension method, bacterial colonies were selected and suspended in a sterile 0.85% (w/v) NaCl. The bacterial inoculum was adjusted to a concentration of 1.5 × 10⁸ CFU/mL by measuring the optical density via a spectrophotometer (OD_600_ = 1.0 for all strains and OD_600_ = 0.8–0.9 for *A. veronii*).

#### Determination of antibacterial activity

The agar disc diffusion method was used to evaluate the antibacterial activity of AgNPs synthesized from algal extracts. Bacterial suspensions were prepared at a concentration of 1.5 × 10⁸ CFU/mL and spread on TSA plates via a sterile cotton swab. Each plate contained six wells with diameters of 20 mm and 8 mm. Test samples of AgNPs at a concentration of 10 mg/mL were added to the wells, while AgNPs synthesized from NaOH were used as the control. Amoxicillin (2 mg/mL) served as the positive control, and 1% (v/v) dimethyl sulfoxide (DMSO) was used as the negative control. Plates were incubated at 30 °C for 20 h before measuring the inhibition zones in millimeters.

#### Determination of minimum inhibitory concentrations (MIC) and minimum bactericidal concentrations (MBC)

The MIC and MBC of AgNPs, which exhibit significant antibacterial activity against fish pathogens and four bacterial strains, were determined using the microdilution method. In sterile 96-well microplates, 100 µL of growth medium, 50 µL of bacterial suspension (1.5 × 10⁶ CFU/mL), and 50 µL of AgNPs at various concentrations (5,000, 2,500, 1,250, 625, 312.5, 156.25, 78.12, 39.06, 19.53, 9.76, 4.88, 2.44, 1.22, 0.61, and 0.31 µg/mL) were added. The negative control consisted of tryptic soy broth (TSB) supplemented with 1% DMSO, whereas the positive control consisted of TSB supplemented with bacterial inoculum. The plates were incubated at 30 °C for fish pathogens and at 37 °C for the other bacterial strains for 24 h. For MBC determination, 100 µL was taken from each well after 24 h and cultured on agar medium for another 24 h at 30 °C for fish pathogens and at 37 °C for the other strains. The MBC was defined as the lowest concentration of AgNPs at which no visible bacterial growth was observed on the TSA plates, indicating a 99.9% reduction in the initial bacterial population. All MIC and MBC determinations were performed in triplicate.

#### Antimycobacterial activity test

The microplate 7H11 agar proportion method^22^ was employed to evaluate the antimycobacterial activity of AgNPs against *M. marinum*. The bacterial suspension was prepared in Middlebrook 7H9 broth supplemented with glycerol and Middlebrook Oleic Albumin Dextrose Catalase (OADC), and its turbidity was adjusted to match McFarland standard No. 1 (approximately 3 × 10⁸ CFU/mL). A volume of 250 µL of the bacterial suspension was added to each of the 12 flat-bottom wells, followed by 250 µL of AgNPs (10 mg/mL) and 1,500 µL of Middlebrook 7H11 supplemented with OADC media into each well. The plates were incubated at 37 °C for 5, 7, 11, or 21 days.

### Statistical analysis

All experiments were performed in triplicate, and the data are expressed as means ± standard deviations. One-way analysis of variance (ANOVA) was performed using SPSS to evaluate differences between groups, with *p* < 0.05 considered statistically significant.

## Results

### Characterization of AgNPs

**Fig. 1:**
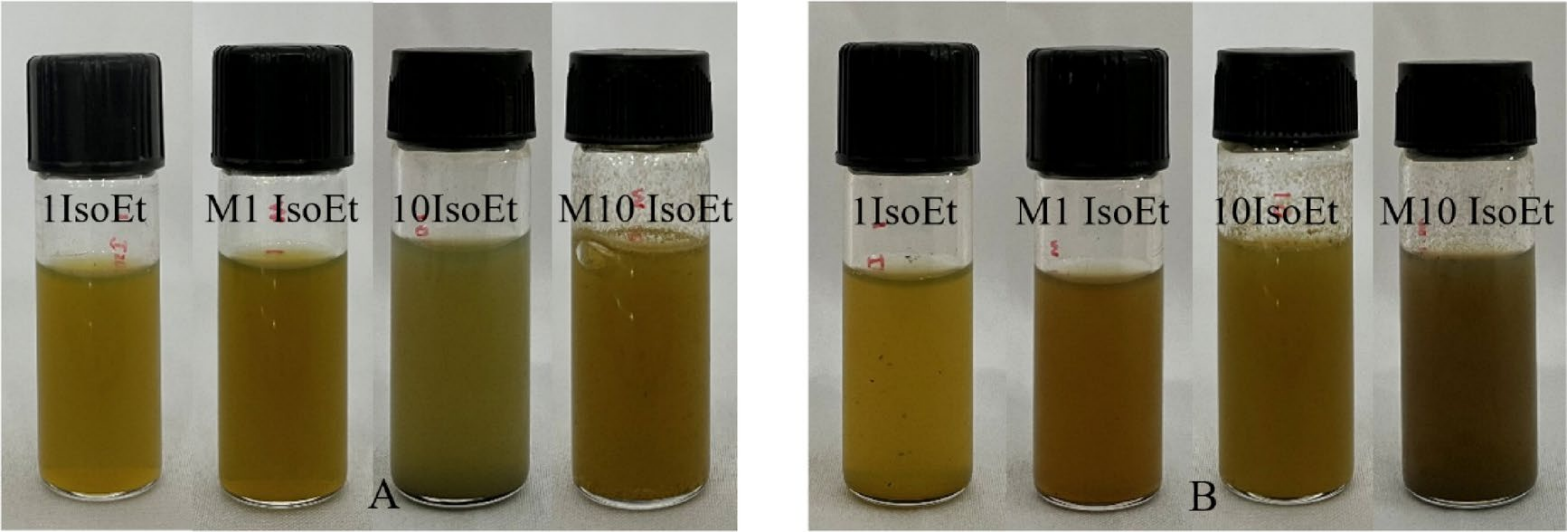
Color change indicating nanoparticle formation in *I. galbana* ethanolic extract-treated AgNO_3_ samples. (**A**) Samples before synthesis and (**B**) samples following synthesis. Sample designations are as follows: 1IsoEt (AgNPs synthesized via the conventional method using 1 mM AgNO_3_), M1 IsoEt (AgNPs synthesized via the microwave-assisted method using 1 mM AgNO_3_) 10IsoEt (conventional method with 10 mM AgNO_3_) and M10 IsoEt (microwave-assisted method with 10 mM AgNO_3_).

**Fig. 2:**
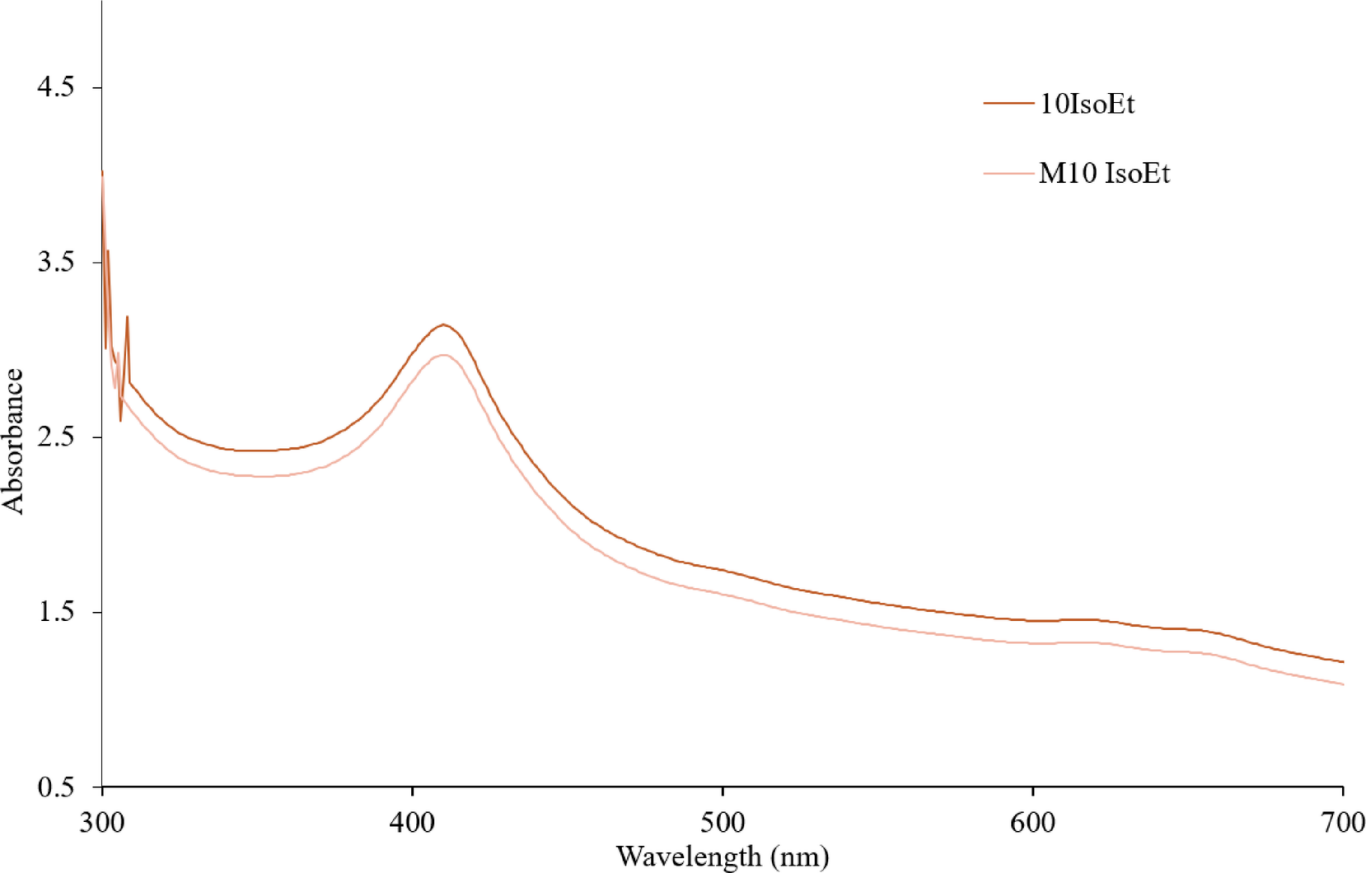
UV-Vis spectra of AgNPs synthesized from AgNO_3_ solutions with *I. galbana* ethanolic extract. The upper line represents 10IsoEt (AgNPs synthesized via conventional stirring with 10 mM AgNO_3_), and the lower line represents M10 IsoEt (AgNPs synthesized via microwave-assisted synthesis with 10 mM AgNO_3_). Both samples were prepared using *I. galbana* ethanolic extract.

**Fig. 3:**
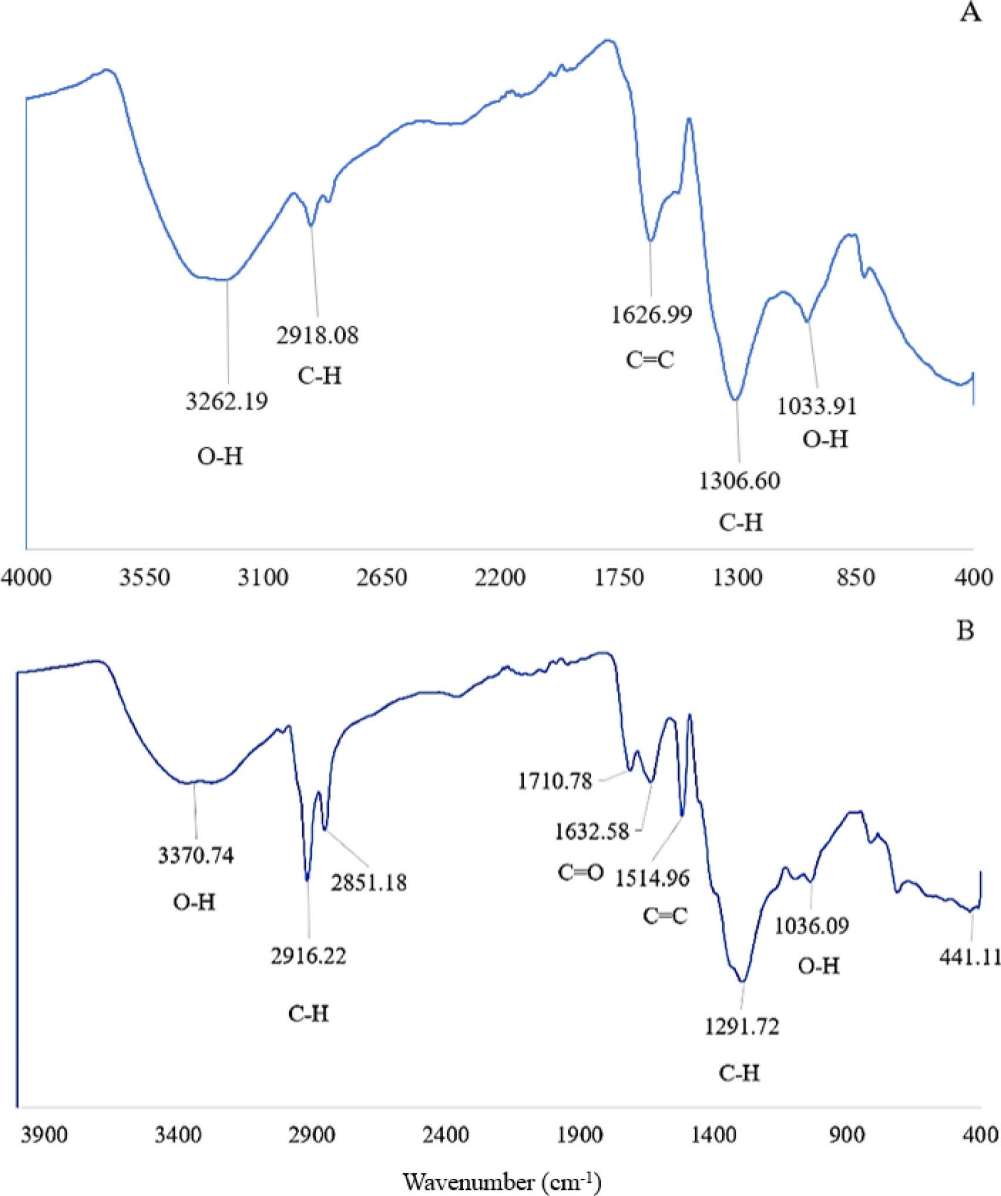
FTIR spectra of M10 IsoEt (**A**) and M10 ChaEt (**B**). M10 IsoEt refers to AgNPs synthesized via microwave-assisted synthesis using 10 mM AgNO_3_ mixed with *I. galbana* ethanolic extract, while M10 ChaEt refers to AgNPs synthesized under the same conditions using *C. calcitrans* ethanolic extract. Individual spectra are also provided separately in the supplementary file.

The synthesis of AgNPs was carried out with 1 mM and 10 mM AgNO_3_ with marine algal extracts in an Erlenmeyer flask at a 1:10 ratio. The reduction of AgNO_3_ was visually confirmed by a color change from pale yellow to dark brown, as shown in Fig. 1. Upon addition of the aqueous AgNO_3_ solution, the marine algal extract became turbid, indicating the initiation of the reaction. The intensity of the brown color increased proportionally over 24 h. The synthesized AgNPs were characterized using UV-Vis spectrophotometry, revealing a peak between 410 and 430 nm for AgNPs produced by both conventional and microwave irradiated marine microalgae at both concentrations. A broader absorption spectrum ranging from 360 to 700 nm was also observed (Fig. 2). The synthesized AgNPs exhibited a characteristic absorption spectrum, suggesting surface plasmon resonance of metallic silver. The yield of AgNPs was quantified, with the 1 mM AgNO_3_ producing approximately 0.004–0.009 g of dry powder and the 10 mM AgNO_3_ yielding approximately 0.063–0.089 g. FTIR spectroscopy of the biosynthesized AgNPs from M10 IsoEt revealed five major bands, each corresponding to different functional groups (Fig. 3A). Peaks were observed at 3262.19 cm^–1^ (O-H stretching of phenols or carboxylic acids), 2918.08 cm^–1^ (C-H stretching of alkenes), 1626.99 cm^–1^ (N-H stretching typical of amides, often found in proteins), 1306.06 cm^–1^ (N = O symmetry stretching of nitro compounds), and 1033.91 cm^–1^ (C-H stretching of alkanes). M10 ChaEt (Fig. 3B) showed strong antibacterial activity next to M10 IsoEt, with results comparable to those of M10 IsoAc. M10 IsoAc exhibited peaks at 3741.79 cm^–1^ (phenol), 3354.51–2917.41 cm^–1^ (aliphatic amines in proteins), 2850.39 cm^–1^ (carboxylic acids), 2075.51 cm^–1^ (silicon compounds), 1641.58 cm^–1^ (alkanes), 1552.93 cm^–1^ (diketones), 1344.89 cm^–1^ (aromatic compounds), and 416.30 cm^–1^ (alkyl halides).

**Fig. 4:**
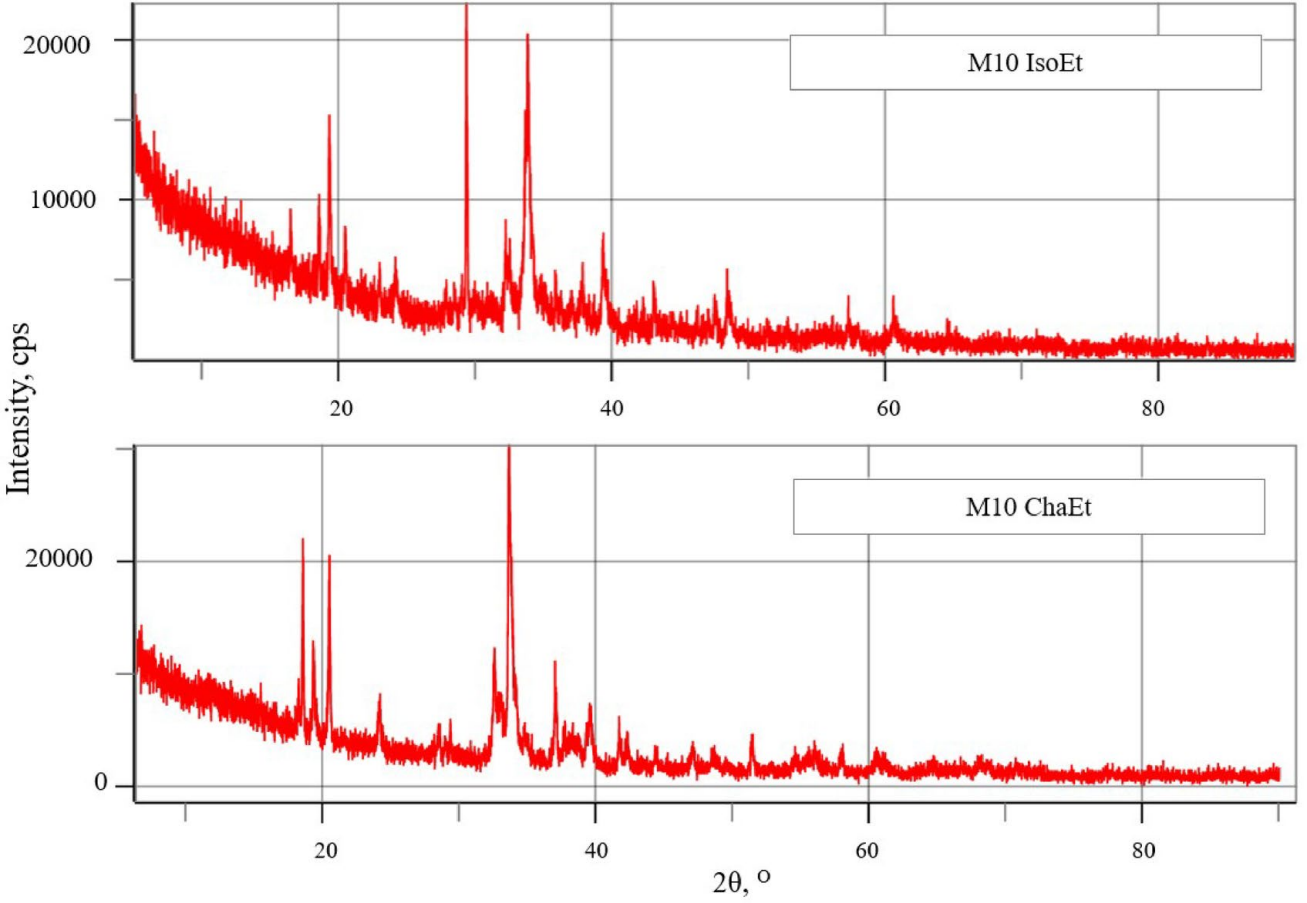
XRD pattern analysis of AgNPs synthesized using microwave-assisted synthesis with 10 mM AgNO_3_ and ethanolic extracts from *I. galbana* (M10 IsoEt) and *C. calcitrans* (M10 ChaEt). M10 IsoEt refers to AgNPs synthesized with *I. galbana* extract, and M10 ChaEt refers to those synthesized with *C. calcitrans* extract. Individual XRD patterns are provided separately in the supplementary file.

XRD analysis was used to assess the crystalline nature of AgNPs produced from *I*. *galbana* and *C*. *calcitrans* ethanolic extracts. The XRD patterns revealed characteristic peaks for AgNPs synthesized with *I. galbana* (Fig. 4), indicating their crystalline structure and small size. Five peaks were observed between 2θ values of 24° and 36° for AgNPs synthesized with M10 IsoEt. The peak at 36° indicated the formation of pure silver (Ag) at the start of the reaction. The XRD spectrum for M10 IsoEt displayed peaks at 2θ values of 27.77°, 32.18°, 38.06°, 46.18°, and 54.78°, corresponding to Ag crystalline planes (111), (200), (220), and (311), respectively. Similarly, M10 ChaEt showed peaks at 27.81°, 32.16°, 34.44°, and 46.19°, corresponding to Ag crystalline planes (111), (200), (203), and (220), respectively. The formation of M10 IsoEt involves coupling reactions with O-H groups from alcohols.

**Fig. 5:**
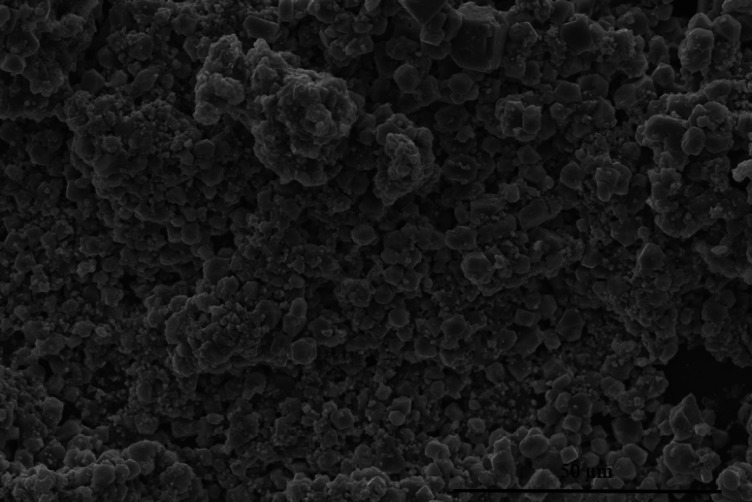
SEM image of AgNPs synthesized as M10 IsoEt. M10 IsoEt refers to AgNPs synthesized via microwave-assisted synthesis using 10 mM AgNO_3_ mixed with *I. galbana* ethanolic extract. Separate figures are provided individually in the supplementary file.

**Fig. 6:**
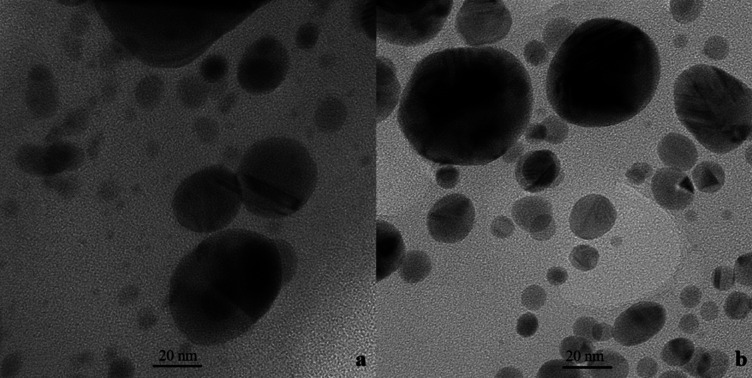
TEM image of AgNPs synthesized using the microwave-assisted method (M10 IsoEt), where 10 mM AgNO_3_ was mixed with *I. galbana* ethanolic extract (**a**), and compared with AgNPs synthesized by the conventional method (**b**). Separate figures are provided individually in the supplementary file.

SEM was employed to examine the morphology of the biosynthesized AgNPs. The SEM images confirmed that AgNPs were crystalline and approximately 100 nm in size (Fig. 5). Further SEM analysis of AgNPs embedded in *Isochrysis galbana* extracts (1IsoEt, M1 IsoEt, 10IsoEt, and M10 IsoEt) revealed particle sizes ranging from 8 to 60 nm. The crystalline nature of the nanoparticles is likely attributed to the presence of capping or stabilizing agents in the extracts. Morphology and particle size were also analyzed using TEM to compare AgNPs synthesized via microwave-assisted and conventional synthesis methods. The synthesis was performed using *I. galbana* extract in combination with 10 mM AgNO_3_, as shown in Fig. 6a (M10 IsoEt) and Fig. 6b (10IsoEt). The observed particle sizes ranged from 5 to 60 nm.

### Antibacterial activity of AgNPs

The antibacterial effects of the AgNPs are summarized in Table 2. This experiment used the agar well diffusion method to compare the effectiveness of two AgNO_3_ concentrations (1 mM and 10 mM) and two synthesis methods (conventional and microwave-assisted) in producing AgNPs from extracts of two marine microalgae via three different solvents (ethanol, hexane, and acetone). Preliminary results indicated that higher concentrations of AgNO_3_ enhanced antibacterial activity, and the microwave-assisted synthesis method further increased efficacy in a dose-dependent manner. The highest antibacterial activity, measured by agar well diffusion, was observed with M10 IsoEt, which inhibited *P. aeruginosa* with a zone of inhibition of 22.13 ± 0.59 mm, followed by *B. subtilis* (19.52 ± 2.43 mm), *E. coli* (16.71 ± 0.81 mm), and *S. aureus* (13.33 ± 0.36 mm).

**Table 2:** Antibacterial activity of bacteria against AgNPs.

Bacteria	Diameter of inhibition zone (mm)				
	*E. coli*	*P* *. aeruginosa*	*A. veronii*	*S. aureus*	*B. subtilis*
1IsoEt	10.04 ± 0.13	17.61 ± 0.48	8.89 ± 0.12	9.22 ± 0.31	10.87 ± 0.07
M1 IsoEt	11.95 ± 0.64	19.71 ± 0.07	8.85 ± 0.16	9.37 ± 0.31	12.71 ± 0.45
10IsoEt	14.37 ± 0.77	20.86 ± 0.12	9.45 ± 0.57	12.26 ± 0.31	12.72 ± 0.29
M10 IsoEt	16.71 ± 0.81	22.13 ± 0.58	10.35 ± 0.16	13.33 ± 0.30	19.52 ± 0.43
1IsoHe	10.00 ± 0.04	17.51 ± 0.25	8.44 ± 0.32	9.62 ± 0.22	10.85 ± 0.12
M1 IsoHe	10.41 ± 0.78	17.59 ± 0.04	8.85 ± 0.01	10.88 ± 0.22	13.56 ± 0.97
10IsoHe	11.76 ± 0.18	18.92 ± 0.09	8.99 ± 0.54	12.03 ± 0.61	14.81 ± 0.91
M10 IsoHe	11.76 ± 0.16	19.80 ± 0.29	10.19 ± 0.35	12.14 ± 0.31	15.24 ± 0.30
1IsoAc	9.93 ± 0.30	17.56 ± 0.21	8.59 ± 0.23	9.78 ± 0.44	12.18 ± 0.27
M1 IsoAc	10.04 ± 0.30	17.98 ± 0.78	8.54 ± 0.19	10.99 ± 0.61	12.20 ± 0.28
10IsoAc	10.81 ± 0.14	18.76 ± 0.09	9.10 ± 0.06	11.83 ± 0.61	13.57 ± 0.48
M10 IsoAc	11.85 ± 0.01	18.98 ± 0.01	9.39 ± 0.47	12.31 ± 0.61	13.78 ± 0.28
1ChaEt	9.94 ± 0.06	17.84 ± 0.21	8.49 ± 0.04	9.56 ± 0.44	10.48 ± 0.25
M1 ChaEt	10.15 ± 0.26	18.15 ± 0.64	9.07 ± 0.09	10.18 ± 0.61	10.33 ± 0.30
10ChaEt	12.11 ± 0.22	19.22 ± 0.11	9.64 ± 0.23	12.06 ± 0.61	14.68 ± 0.24
M10 ChaEt	12.59 ± 0.18	20.02 ± 0.63	11.17 ± 0.47	13.23 ± 0.31	16.66 ± 0.45
1ChaHe	9.96 ± 0.20	17.54 ± 0.47	8.78 ± 0.19	9.34 ± 0.44	10.11 ± 0.84
M1 ChaHe	10.11 ± 0.93	17.71 ± 0.45	8.87 ± 0.01	10.31 ± 0.22	10.73 ± 0.04
10ChaHe	11.36 ± 0.77	18.92 ± 0.44	8.89 ± 0.05	11.64 ± 0.61	13.12 ± 0.07
M10 ChaHe	12.23 ± 0.58	19.14 ± 0.52	9.30 ± 0.18	11.91 ± 0.61	15.12 ± 0.38
1ChaAc	9.92 ± 0.09	17.68 ± 0.30	8.69 ± 0.45	9.91 ± 0.22	13.12 ± 0.96
M1 ChaAc	10.32 ± 0.49	18.09 ± 0.95	8.93 ± 0.01	10.14 ± 0.22	14.20 ± 0.42
10ChaAc	11.82 ± 0.48	18.70 ± 0.49	9.42 ± 0.70	11.79 ± 0.31	15.15 ± 0.25
M10 ChaAc	12.45 ± 0.33	18.88 ± 0.77	10.6 ± 0.21	12.06 ± 0.61	16.48 ± 0.63
1 mM AgNPs	7.10 ± 0.41	17.50 ± 0.29	8.37 ± 0.11	9.13 ± 0.30	9.66 ± 0.38
10 mM AgNPs	7.70 ± 0.78	18.69 ± 0.59	9.02 ± 0.11	11.78 ± 0.11	11.67 ± 0.99
Positive control	19.72 ± 0.24	7.14 ± 0.17	8.49 ± 0.40	9.73 ± 0.97	10.98 ± 0.63
Negative control	0	0	0	0	0

**Fig. 7:**
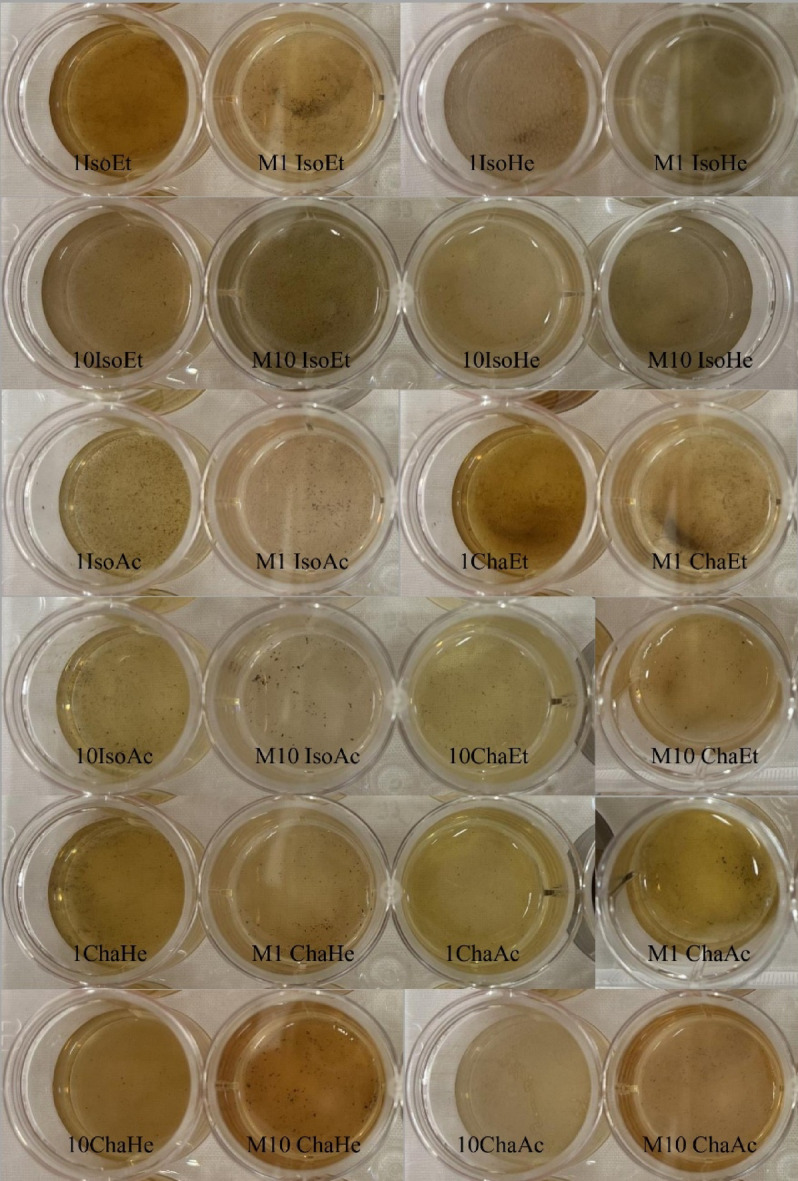
Antimycobacterial activity of various AgNPs against *Mycobacterium marinum* using the Microplate 7H11 agar proportional method. Clear inhibition zones were observed around wells containing AgNPs synthesized by different methods and extracts, indicating their antimycobacterial effectiveness.

In this study, biologically synthesized AgNPs were also evaluated against clinical bacterial isolates using a microplate Alamar blue assay. The results showed that the concentration of AgNO_3_ had a significant effect on bacterial inhibition, with both synthesis methods and the type of marine microalgal extract influencing the outcome. Notably, AgNPs synthesized via microwave irradiation at a concentration of 10 mM demonstrated the strongest antibacterial effect. Additionally, ethanol-extracted algae produced superior antibacterial activity compared to other extraction methods. All clinical isolates were inhibited within a MIC range of 0.31–625 µg/mL and an MBC range of 0.31-1,250 µg/mL, as shown in Table 3. The MIC values of the ethanolic extract of *I. galbana*-synthesized AgNPs against *E. coli*, *P. aeruginosa*, and *S. aureus* were 0.31 ± 0.00 µg/mL, whereas those against *A. veronii* and *B. subtilis* were 16.27 ± 5.64 µg/mL and 0.31 ± 0.00 µg/mL, respectively. The antimycobacterial activity of the AgNPs was evaluated against *M. marinum* using the microplate 7H11 agar proportional method. All AgNPs samples inhibited the growth of *M. marinum*, even after 21 days of incubation (Fig. 7).

**Table 3:** MIC and MBC of bacteria against AgNPs (µg).

Sample	*E. coli*		*P. aeruginosa*		*A. veronii*		*S. aureus*		*B. subtilis*	
	MBC	MIC	MBC	MIC	MBC	MIC	MBC	MIC	MBC	MIC
1 IsoEt	9.76 ± 0.00	0.61 ± 0.00	2.44 ± 0.00	0.31 ± 0.00	78.12 ± 0.00	39.06 ± 0.00	9.76 ± 0.00	0.31 ± 0.00	9.76 ± 0.00	0.31 ± 0.00
M 1 IsoEt	9.76 ± 0.00	0.61 ± 0.00	1.22 ± 0.00	0.31 ± 0.00	78.12 ± 0.00	39.06 ± 0.00	4.88 ± 0.00	0.31 ± 0.00	2.44 ± 0.00	0.31 ± 0.00
10 IsoEt	9.76 ± 0.00	0.31 ± 0.00	0.31 ± 0.00	0.31 ± 0.00	39.06 ± 0.00	16.27 ± 5.64	4.88 ± 0.00	0.31 ± 0.00	2.44 ± 0.00	0.31 ± 0.00
M 10 IsoEt	4.07 ± 1.41	0.31 ± 0.00	0.31 ± 0.00	0.31 ± 0.00	39.06 ± 0.00	16.27 ± 5.64	0.61 ± 0.00	0.31 ± 0.00	0.31 ± 0.00	0.31 ± 0.00
1 IsoHe	4.88 ± 0.00	0.61 ± 0.00	4.88 ± 0.00	0.61 ± 0.00	156.5 ± 0.00	78.12 ± 0.00	9.76 ± 0.00	1.22 ± 0.00	4.88 ± 0.00	0.61 ± 0.00
M 1 IsoHe	4.88 ± 0.00	1.22 ± 0.00	1.22 ± 0.00	0.31 ± 0.00	78.12 ± 0.00	39.06 ± 0.00	4.88 ± 0.00	1.22 ± 0.00	4.88 ± 0.00	0.61 ± 0.00
10 IsoHe	4.88 ± 0.00	1.22 ± 0.00	1.22 ± 0.00	0.31 ± 0.00	39.06 ± 0.00	19.53 ± 0.00	2.44 ± 0.00	0.61 ± 0.00	1.22 ± 0.00	0.31 ± 0.00
M 10 IsoHe	1.22 ± 0.00	0.31 ± 0.00	1.22 ± 0.00	0.31 ± 0.00	39.06 ± 0.00	19.53 ± 0.00	1.22 ± 0.00	0.31 ± 0.00	1.22 ± 0.00	0.31 ± 0.00
1 IsoAc	9.76 ± 0.00	2.44 ± 0.00	2.44 ± 0.00	0.61 ± 0.00	312.5 ± 0.00	156.5 ± 0.00	4.88 ± 0.00	2.44 ± 0.00	2.44 ± 0.00	0.61 ± 0.00
M 1 IsoAc	4.88 ± 0.00	2.44 ± 0.00	1.22 ± 0.00	0.61 ± 0.00	156.5 ± 0.00	78.12 ± 0.00	2.44 ± 0.00	0.61 ± 0.00	1.22 ± 0.00	0.61 ± 0.00
10 IsoAc	2.44 ± 0.00	0.61 ± 0.00	1.22 ± 0.00	0.61 ± 0.00	78.12 ± 0.00	39.06 ± 0.00	1.22 ± 0.00	0.61 ± 0.00	1.22 ± 0.00	0.61 ± 0.00
M 10 IsoAc	1.22 ± 0.00	0.61 ± 0.00	1.22 ± 0.00	0.61 ± 0.00	78.12 ± 0.00	39.06 ± 0.00	1.22 ± 0.00	0.61 ± 0.00	0.61 ± 0.00	0.31 ± 0.00
1 ChaEt	9.76 ± 0.00	2.44 ± 0.00	4.88 ± 0.00	0.61 ± 0.00	1250 ± 0.00	625 ± 0.00	9.76 ± 0.00	2.44 ± 0.00	4.88 ± 0.00	0.61 ± 0.00
M 1 ChaEt	9.76 ± 0.00	2.44 ± 0.00	1.22 ± 0.00	0.61 ± 0.00	1250 ± 0.00	625 ± 0.00	4.88 ± 0.00	0.61 ± 0.00	1.22 ± 0.00	0.31 ± 0.00
10 ChaEt	4.88 ± 0.00	2.44 ± 0.00	1.22 ± 0.00	0.61 ± 0.00	625 ± 0.00	312.5 ± 0.00	2.44 ± 0.00	0.61 ± 0.00	0.61 ± 0.00	0.31 ± 0.00
M 10 ChaEt	2.44 ± 0.00	1.02 ± 0.35	0.61 ± 0.00	0.31 ± 0.00	625 ± 0.00	312.5 ± 0.00	1.22 ± 0.00	0.61 ± 0.00	0.31 ± 0.00	0.31 ± 0.00
1 ChaHe	4.88 ± 0.00	2.44 ± 0.00	4.88 ± 0.00	2.44 ± 0.00	1250 ± 0.00	625 ± 0.00	9.76 ± 0.00	2.44 ± 0.00	4.88 ± 0.00	2.44 ± 0.00
M 1 ChaHe	4.88 ± 0.00	2.44 ± 0.00	2.44 ± 0.00	0.61 ± 0.00	1250 ± 0.00	625 ± 0.00	4.88 ± 0.00	1.22 ± 0.00	2.44 ± 0.00	0.61 ± 0.00
10 ChaHe	4.88 ± 0.00	2.44 ± 0.00	2.44 ± 0.00	0.61 ± 0.00	1250 ± 0.00	625 ± 0.00	4.88 ± 0.00	0.61 ± 0.00	1.22 ± 0.00	0.31 ± 0.00
M 10 ChaHe	2.44 ± 0.00	1.22 ± 0.00	1.22 ± 0.00	0.31 ± 0.00	78.12 ± 0.00	39.06 ± 0.00	4.88 ± 0.00	0.61 ± 0.00	1.22 ± 0.00	0.31 ± 0.00
1 ChaAc	4.88 ± 0.00	0.61 ± 0.00	4.88 ± 0.00	0.61 ± 0.00	312.5 ± 0.00	156.5 ± 0.00	4.88 ± 0.00	1.22 ± 0.00	4.88 ± 0.00	0.31 ± 0.00
M 1 ChaAc	2.44 ± 0.00	0.61 ± 0.00	2.44 ± 0.00	0.61 ± 0.00	156.5 ± 0.00	78.12 ± 0.00	4.88 ± 0.00	1.22 ± 0.00	4.88 ± 0.00	0.31 ± 0.00
10 ChaAc	2.44 ± 0.00	0.61 ± 0.00	0.61 ± 0.00	0.31 ± 0.00	78.12 ± 0.00	39.06 ± 0.00	4.88 ± 0.00	0.61 ± 0.00	2.44 ± 0.00	0.31 ± 0.00
M 10 ChaAc	2.44 ± 0.00	0.61 ± 0.00	0.61 ± 0.00	0.31 ± 0.00	78.12 ± 0.00	39.06 ± 0.00	4.88 ± 0.00	0.61 ± 0.00	1.22 ± 0.00	0.31 ± 0.00
1 mM AgNPs	78.12 ± 0.00	2.44 ± 0.00	156.5 ± 0.00	1.22 ± 0.00	156.5 ± 0.00	78.12 ± 0.00	39.06 ± 0.00	4.88 ± 0.00	19.53 ± 0.00	2.44 ± 0.00
10 mM AgNPs	4.88 ± 0.00	2.44 ± 0.00	9.76 ± 0.00	1.22 ± 0.00	78.12 ± 0.00	39.06 ± 0.00	19.53 ± 0.00	1.22 ± 0.00	9.76 ± 0.00	1.22 ± 0.00
Positive control	2500 ± 0.00	1250 ± 0.00	2500 ± 0.00	1250 ± 0.00	1250 ± 0.00	312.5 ± 0.00	1250 ± 0.00	312.5 ± 0.00	625 ± 0.00	156.5 ± 0.00

## Discussion

The visual observation of the biosynthesized AgNPs revealed a color change in the solution from yellow-green to dark brown. This was the first significant indication of AgNPs synthesis from each marine microalgal extract using both conventional and microwave irradiation methods with 1 mM and 10 mM AgNO_3_. Nanoparticle formation occurred when the initial concentration of reactants was within an optimal range and was influenced by bioactive compounds in the natural extracts, which facilitated radical reactions and acted as stabilizing agents. The ratio of extract to AgNO_3_ significantly affected the size and morphology of the AgNPs. Upon the addition of the extract to AgNO_3_, a reduction reaction was initiated, and indicated by a color change. As the solution darkened, the extent of nanoparticle formation increased. Both the concentration of AgNO_3_ and the compositional variability of the extracts influenced the reaction kinetics. UV-Vis spectroscopy was employed to confirm and monitor the progress of nanoparticle synthesis. UV-Vis analysis revealed an absorption peak in the range of 410–430 nm in all samples, regardless of variations in the extracts and AgNO_3_ concentrations. No significant differences were observed in the peak positions among the samples. However, the microwave-assisted synthesis method resulted in AgNPs with lower absorbance compared to those synthesized using the conventional method. Our AgNPs exhibited strong absorption, consistent with previous studies that reported peak absorbance within the 410–430 nm range. In general, the absorbance of AgNPs is largely influenced by their shape and size^23^. Decrease or different peak areas suggests the influence of biomolecules present in the synthesis medium on the reaction process, emphasizing how the selection of synthesis components affects the optical properties of the resulting nanoparticles^24^.

SEM imaging revealed the crystalline shapes of the AgNPs, with particle sizes ranging from 8 to 60 nm. TEM analysis provided more detailed visualization, showing particle sizes between 5 and 60 nm. There was no significant difference in particle size between nanoparticles synthesized by microwave-assisted and conventional methods. These findings differ from those reported by Torabfam and Yűce^23^, who synthesized AgNPs using *C. vulgaris* extract and 1 mM AgNO_3_ with microwave assistance, yielding nanoparticles of 1–50 nm in size. Similarly, Suja et al.^24^ reported *I. galbana*-synthesized AgNPs with spherical shapes and sizes ranging from 157.3 to 183.8 nm. In this study, AgNPs with optimal antibacterial activity were selected for further structural analysis, including SEM, TEM, XRD, and FTIR, to reduce energy consumption costs.

XRD analysis was performed to examine the crystalline structure of AgNPs biosynthesized under optimal conditions using an aqueous algal extract. The XRD pattern showed characteristic peaks corresponding to crystalline Ag (111, 200, 220, and 311), which is consistent with the work of Vinayagam et al.^25^. Interestingly, when different algal extracts were used, variations in peak intensities and widths were observed, suggesting differences in particle size, degree of crystallinity, and possibly capping effects from bioactive compounds present in the extracts. These differences suggest that the specific phytochemical composition of each algal extract influences the growth and stabilization of AgNPs, as well as their crystalline structure. Additionally, microwave-assisted synthesis contributed to sharper peaks, signifying higher crystallinity. A few or low intensity peaks were observed, which may be attributed to the crystallization of organic compounds from the algal extract on the Ag surface, functioning as capping agents^25^.

FTIR analysis revealed various functional groups in each sample, with broad absorption peaks associated with O-H, N-H, and C-O stretching vibrations. The results revealed differences in peak positions between samples, with different solvent extracts yielding varying results. For example, Merin et al.^26^ synthesized AgNPs using 10 mM AgNO_3_ with *C. vulgaris* and *C. calcitrans*, finding functional groups such as ethers and aromatic compounds. This variability in shape and size may be attributed to different extraction solvents and synthesis methods, as well as different algal types and cultivation methods, all of which affect the functional groups and chemical components detected. Previous studies have described the adsorption and stabilization of AgNPs by microalgal extracts as a process driven by the coordination of carbonyl groups and the transfer of nearby electrons to the AgNPs^26^. The presence of functional groups has a critical role in the stabilization of AgNPs. These functional groups likely act as capping agents, enhancing the stability of the nanoparticles by preventing aggregation through steric hindrance and electrostatic repulsion. This FTIR analysis confirmed the involvement of main functional groups in facilitating the reduction of Ag⁺ to AgNPs and stabilizing the resulting nanoparticles. These findings align with previous studies, which reported similar functional groups, including amines, phenols, ethers, and aromatic rings, participation in nanoparticle synthesis and stabilization mechanisms^15^. Additionally, the variability in functional group composition among different solvent extracts highlights their influence on nanoparticle morphology and stabilization.

This research highlights that particle morphology, shape, and size are crucial factors in AgNPs synthesis. During their biosynthesis, phytochemical compounds from algal extracts reduce Ag^+^ to AgNPs while also acting as stabilizing agents. The algal extracts contain surfactants that reduce the surface tension between AgNPs and the solution, preventing aggregation and controlling size and shape of the nanoparticles. For example, *I. galbana* extracts contain lipids, polyunsaturated fatty acids, and carbohydrates, which function as surfactants^27^. Similarly, fucoxanthin, which is found in *C. calcitrans*, also acts as a surfactant that stabilizes nanoparticles via encapsulation^28,29^. These compounds ensure the formation of smaller, well-dispersed nanoparticles, which are essential characteristics in AgNPs synthesis. The synthesis process of AgNPs is influenced by several factors, with the extract playing a pivotal role. Algal extracts act as both reducing and stabilizing agents, facilitating the formation of stable and smaller AgNPs, which enhances their antimicrobial activity. AgNPs synthesized with extracts have a larger surface area, improving their ability to penetrate microbial cells and disrupt essential cellular functions. Furthermore, the antimicrobial properties of compounds from the extract that remain on the nanoparticle surface are enhanced, making extract-synthesized AgNPs highly effective antimicrobial agents for various applications.

Research findings indicate that AgNPs synthesized via the microwave-assisted method exhibit superior antibacterial activity compared to those produced by the conventional method. Among the tested concentrations, AgNPs synthesized with 10 mM AgNO_3_ showed greater bacterial inhibition than those synthesized with 1 mM AgNO_3_. In antibacterial activity tests via the agar well diffusion method, the M10 IsoEt sample showed the highest inhibition, followed by M10 ChaEt and M10 IsoAc. Gram-negative bacteria (*P. aeruginosa* and *E. coli*) were more susceptible to inhibition than the Gram-positive bacterium *S. aureus.* However, *B. subtilis*, a Gram-positive strain, exhibited greater sensitivity than *E. coli*.

In previous research, *Dunaliella salina* extract was used to synthesize AgNPs with 1 mM AgNO_3_, which resulted in consistent bacterial inhibition^30^. In this study, M10 ChaEt exhibited stronger inhibition against Gram-negative bacterium *A. veronii*. These findings suggest that different microalgal extracts exert specific effects depending on the bacterial strain. This research is consistent with the previous work by Annamalai and Nallamuthu^31^, who reported that different concentrations of AgNO_3_ yielded varied bacterial inhibition zones, with 0.5 mM AgNO_3_ resulting in the greatest inhibition of *B. subtilis* and *E. coli*. Bacterial inhibition tests based on inhibition zone measurements revealed no significant overall difference between Gram-positive and Gram-negative bacteria. However, *P. aeruginosa* showed the greatest susceptibility to inhibition. This finding is consistent with the results reported by Ouardy et al.^32^, who studied the antibacterial effects of AgNPs synthesized from *Parachlorella kesseleri* and *Cyclotella* spp. In their study, nanoparticles synthesized using 1 mM AgNO_3_ resulted in lower MIC values. In contrast, our study employed 10 mM AgNO_3_ and achieved significantly enhanced antibacterial activity, with a MIC as low as 0.31 µg/mL.

The size, shape, surface characteristics of nanoparticles, overall surface area, type of coating, and rate of Ag⁺ ion generation significantly influence their antibacterial mechanisms^7^. Nanoparticles are typically between 1 and 100 nm penetrate bacterial cell walls more effectively and provide a higher surface area for interactions^30^. Positively charged surfaces enhance membrane disruption and promote reactive oxygen species (ROS) generation, damaging bacterial components^6^. Surface functionalization with microalgal extracts improves bacterial targeting and delivery of active compounds. Tailoring these properties optimizes antibacterial efficiency. This study demonstrated that increasing the AgNO_3_ concentration to 10 mM enhances antibacterial activity. AgNPs exhibited a wider inhibition zone, especially against gram-positive bacteria. The antibacterial mechanisms of AgNPs include bioactive ion release, disruption of metabolic pathways, generation of ROS, alteration of cell walls and membranes, and inhibition of bacterial DNA replication^7^. The microwave-assisted synthesis technique ensures uniform heating and accelerates nanoparticle production, improving bacterial inhibition. This approach is promising for applications in aquaculture, particularly in controlling pathogens such as *M. marinum*, a non-tuberculosis pathogen in betta fish.

This research presents the first report of AgNPs synthesized from *I. galbana* and *C. calcitrans* via microwave irradiation to inhibit fish pathogenic bacteria. These findings show that AgNPs effectively inhibit *M. marinum* growth, suggesting potential applications in aquaculture. Further investigations are needed to determine the optimal AgNO_3_ concentration for various environmental conditions, which could contribute to the development of efficient and sustainable aquaculture practices.

## Conclusions

This study explored the biosynthesis of AgNPs via various extracts from marine microalgae. The AgNPs were characterized through multiple techniques, including UV-Vis spectroscopy, XRD, FTIR, SEM, and TEM. These analyses confirmed that the synthesized AgNPs exhibited significant antibacterial activity. The nanoparticles demonstrated potent antibacterial effects against several human and fish pathogens, including *S. aureus*, *B. subtilis*, *E. coli*, *A. veronii*, and *M. marinum*. Among the six extracts from two different microalgal species and from different synthesis methods, the most notable antibacterial activity was observed against these pathogens. The use of natural marine microalgal extracts in nanoparticle synthesis, particularly through microwave irradiation, offers significant advantages. This method reduces synthesis time while enhancing antibacterial effectiveness due to rapid and uniform heating, which improves particle dispersion and stability. Additionally, the antibacterial efficacy of the synthesized nanoparticles is influenced by the concentration of AgNO_3_, highlighting the importance of optimizing precursor concentrations for maximum performance. Future research could investigate the use of other microalgal species, different AgNO_3_ concentrations, and alternative synthesis methods to further assess their antibacterial effects against a wider range of pathogens in aquaculture. These findings could contribute to the development of new products aimed at preventing and treating various diseases in the aquaculture industry.

## Electronic supplementary material

Below is the link to the electronic supplementary material.

Supplementary Material 1

## Data Availability

The data supporting the findings of this study are available from the corresponding author upon request. Additionally, the data have been deposited in GenBank, including the weblink and accession number LC853089 and LC853090.
