# Supplementary material for: Microwave-assisted biosynthesis of silver nanoparticles using two marine microalgal extracts and their antimycobacteriosis activity against bacteria isolated from Betta splendens
Source: Sci Rep. 2025 May 1;15:15315. doi: 10.1038/s41598-025-00128-w (PMC12045969; doi:10.1038/s41598-025-00128-w)
Supplement: Supplementary file 1 — Supplementary Material 1 [file 41598_2025_128_MOESM1_ESM.docx]

Supplementary Information

**Microwave-assisted biosynthesis of silver nanoparticles using two marine microalgal extracts and their antimycobacteriosis activity against bacteria isolated from *Betta splendens***

Piyapan Manklinniam, Saranya Phunpruch, Aparporn Sakulkalavek, Rachsak Sakdanuphab, Worakrit Worananthakij*

*Corresponding authors. E-mail: worakrit.wo@kmitl.ac.th

The file includes:

Supplementary Figures 1 to 4

Supplementary Figure 1


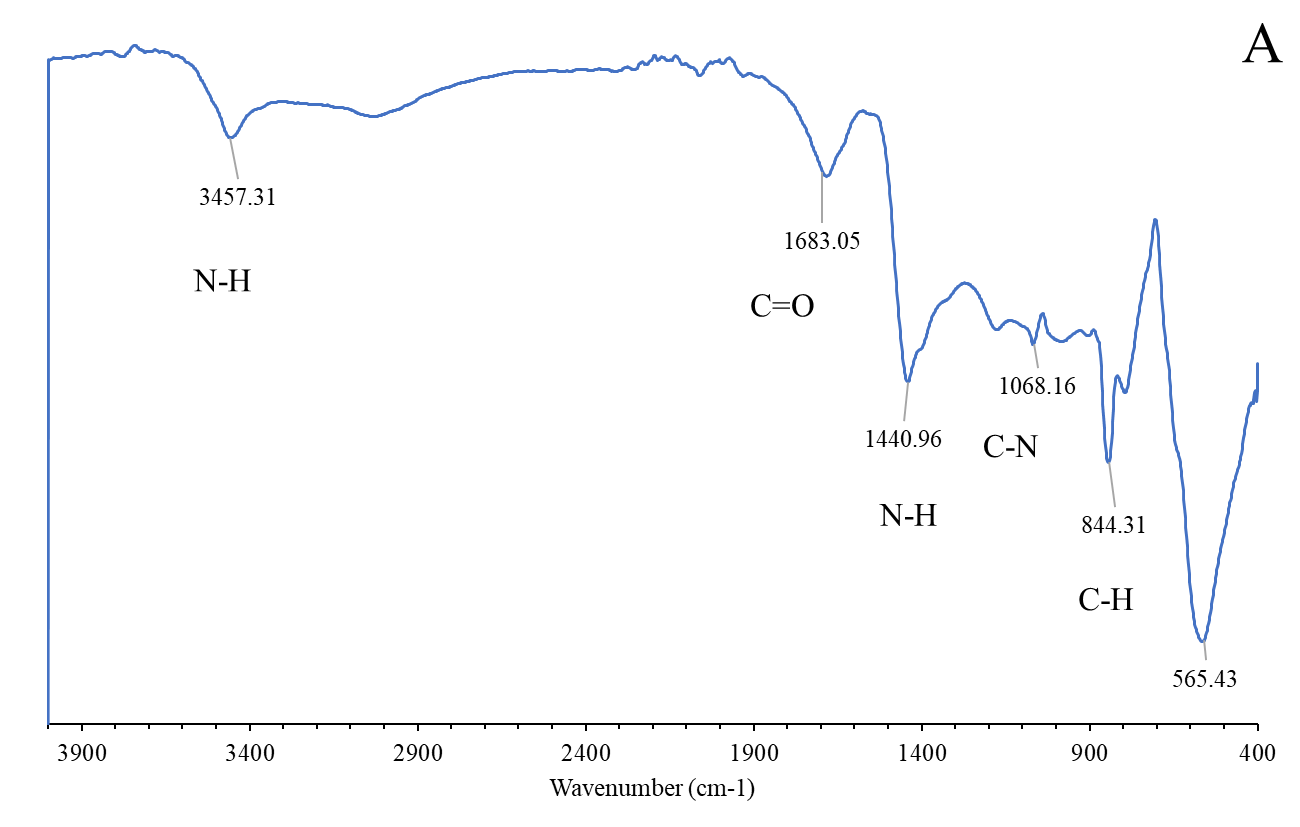

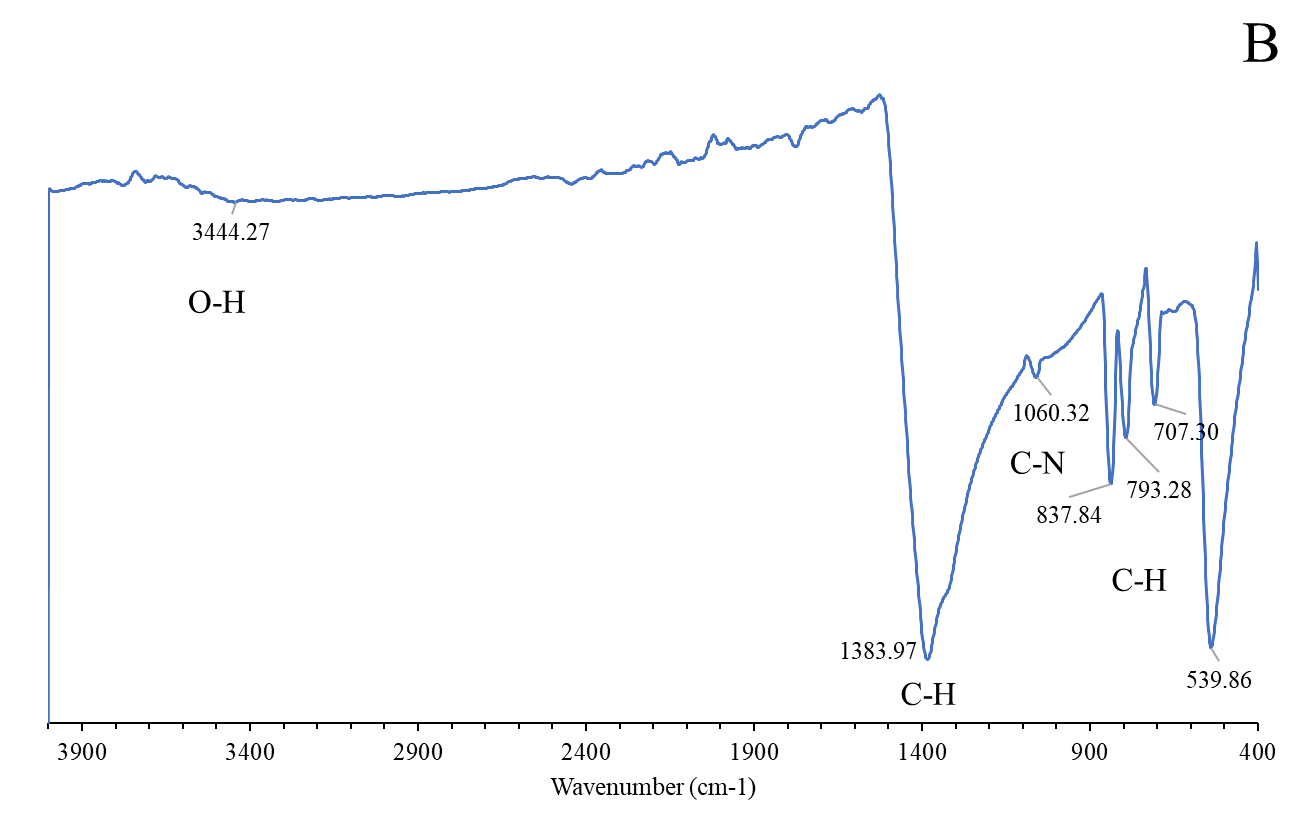

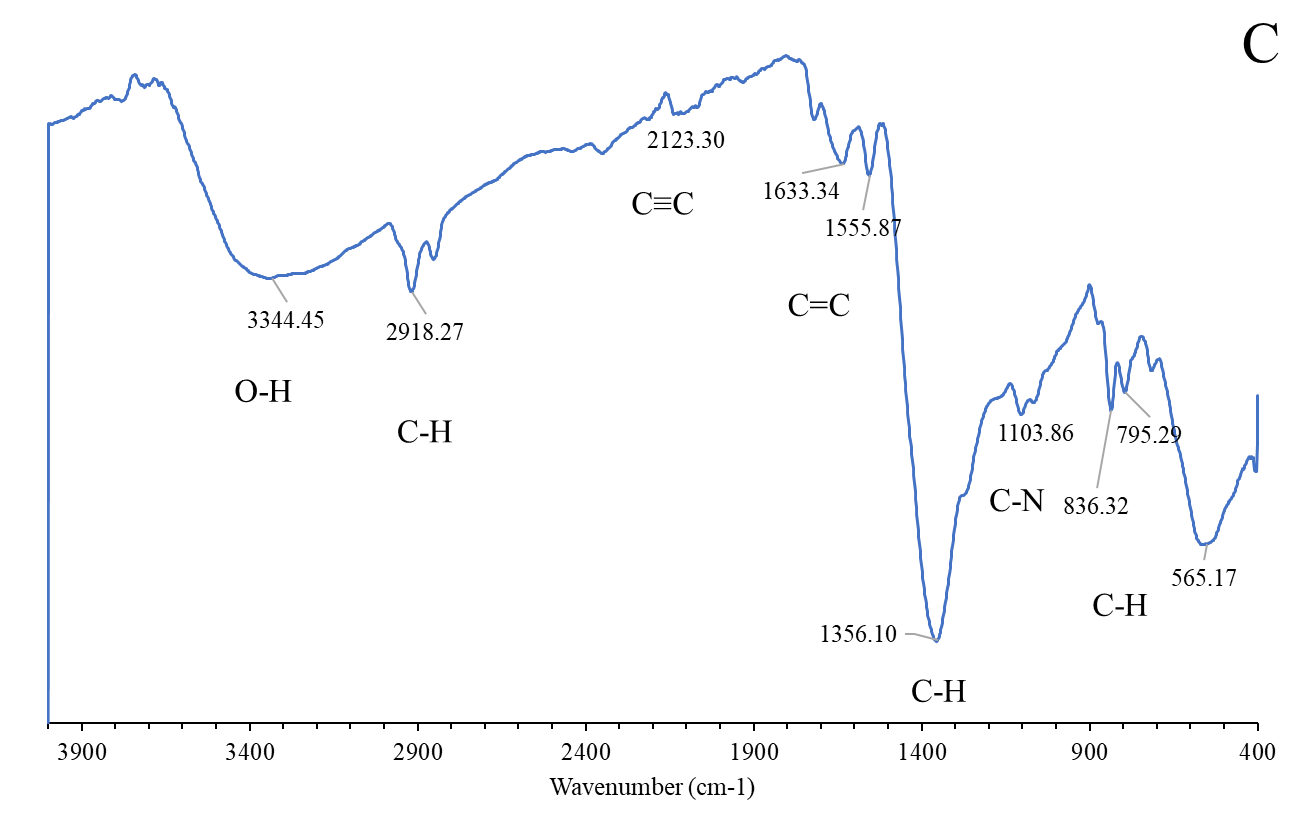

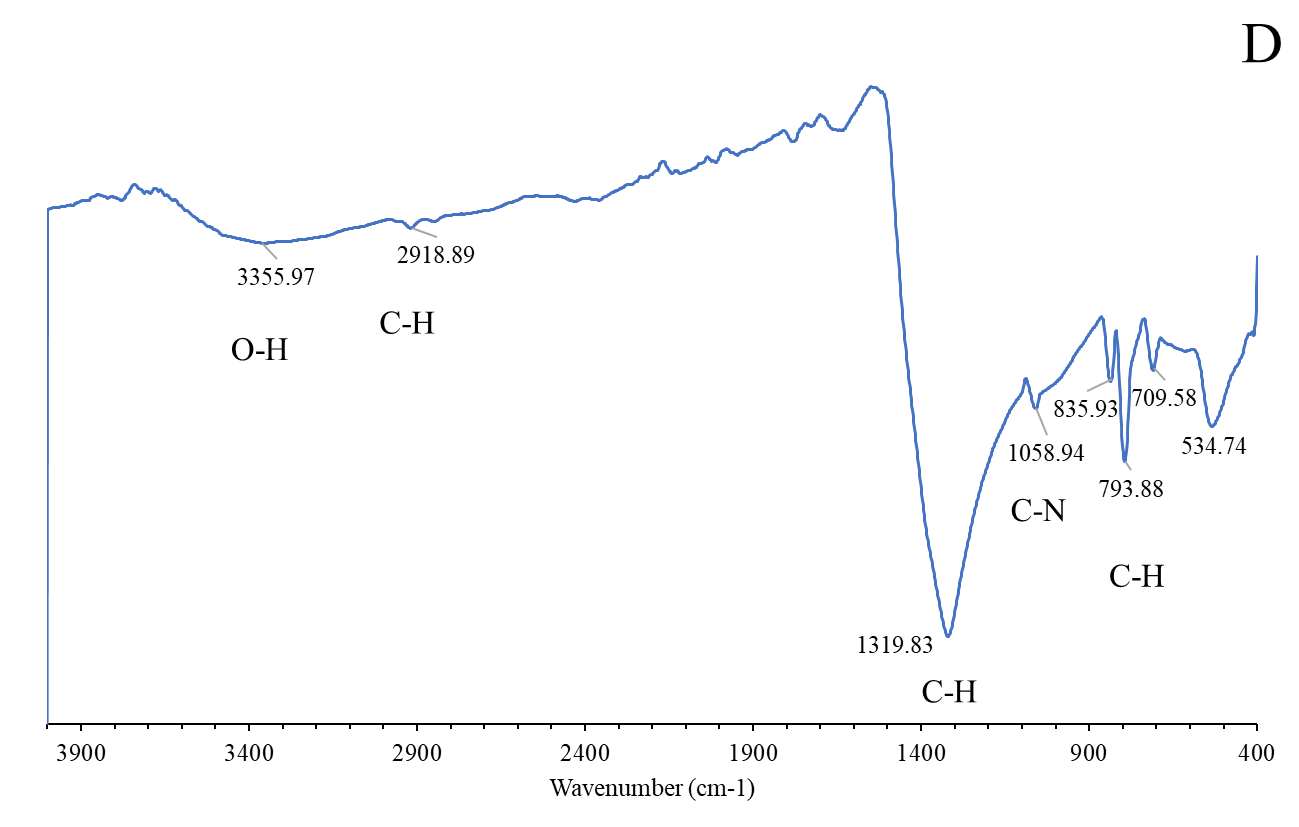


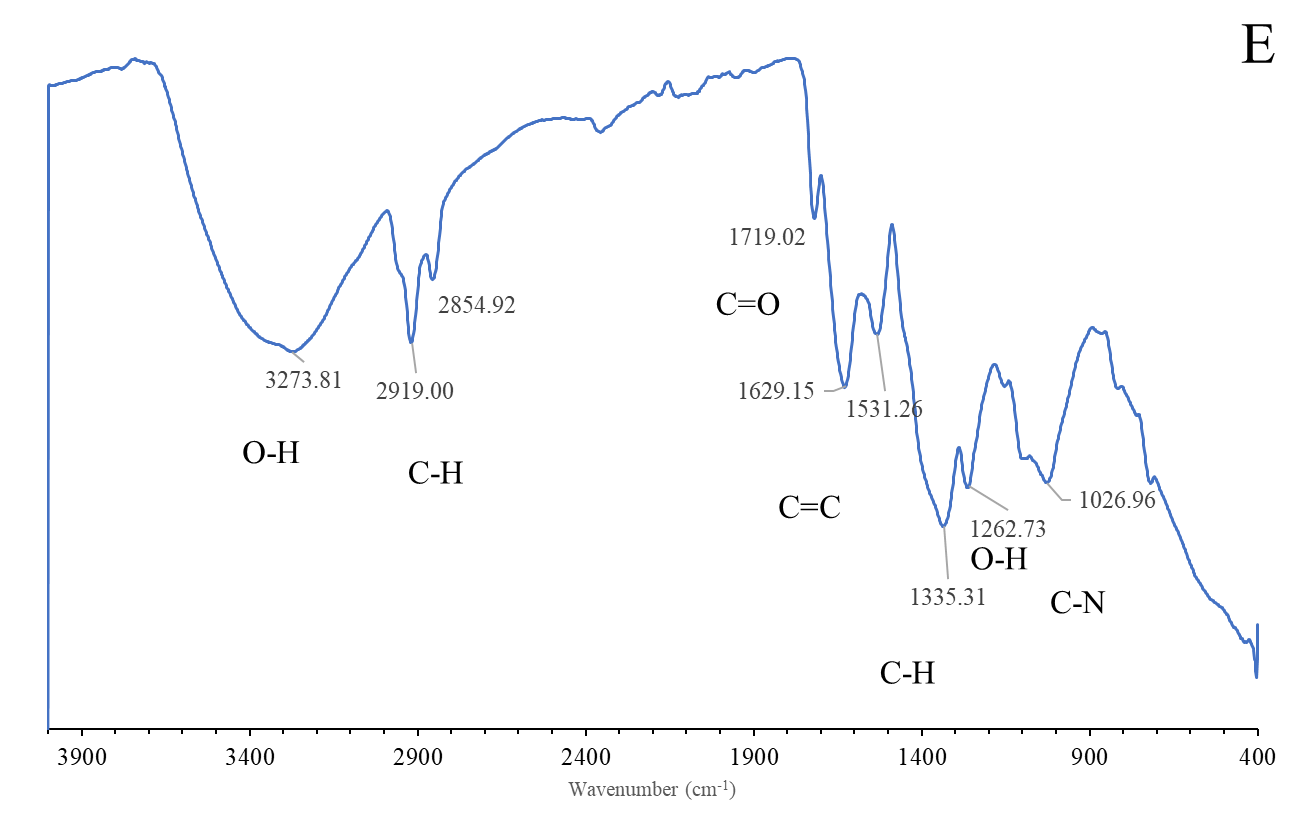

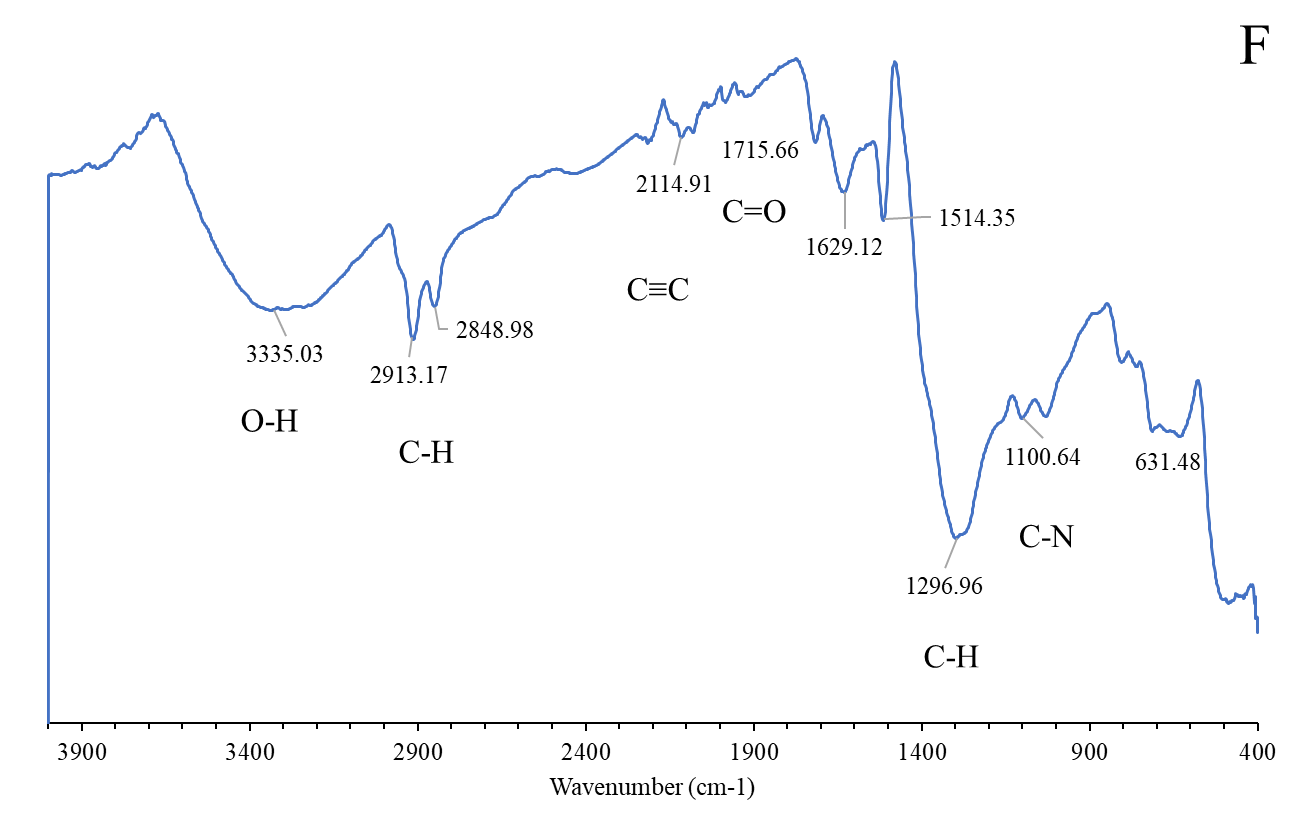


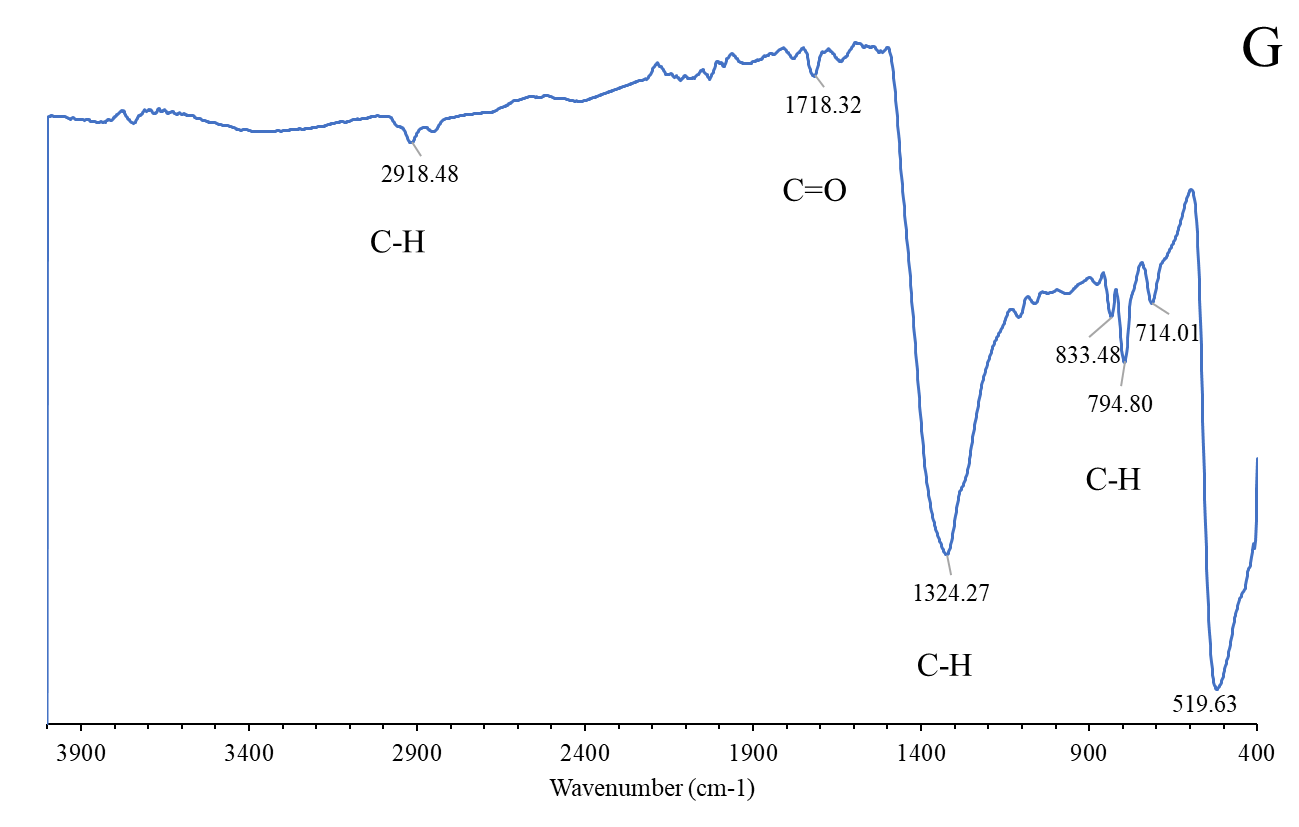


**Supplementary Figure 1: FTIR spectra of AgNPs synthesis. A** 1 mM AgNPs is AgNPs synthesized by the conventional method with 1 mM AgNO_3_ mixed with 0.1 M NaOH. **B** 10 mM AgNPs is AgNPs synthesized by the conventional method with 10 mM AgNO_3_ mixed with 0.1 M NaOH. **C** 1IsoEt is AgNPs synthesis by conventional method with 1 mM AgNO_3_ mixed with *I. galbana* ethanolic extract. **D** 10IsoEt is AgNPs synthesis by conventional method with 10 mM AgNO_3_ mixed with *I. galbana* ethanolic extract. **E** M1 IsoEt is AgNPs synthesis by microwave-assisted synthesis with 1 mM AgNO_3_ mixed with *I. galbana* ethanolic extract. **F** M10 ChaHe is AgNPs synthesis by microwave-assisted synthesis with 10 mM AgNO_3_ mixed with *C. calcitrans* hexane extract. **G** M10 IsoHe is AgNPs synthesis by microwave-assisted synthesis with 10 mM AgNO_3_ mixed with *I. galbana* hexane extract.

Supplementary Figure 2


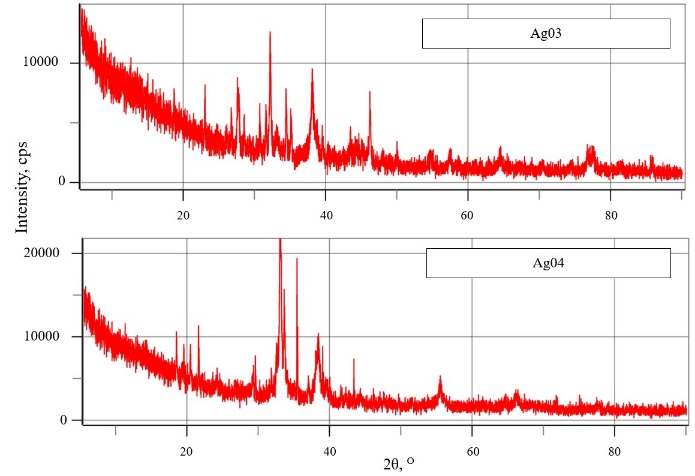

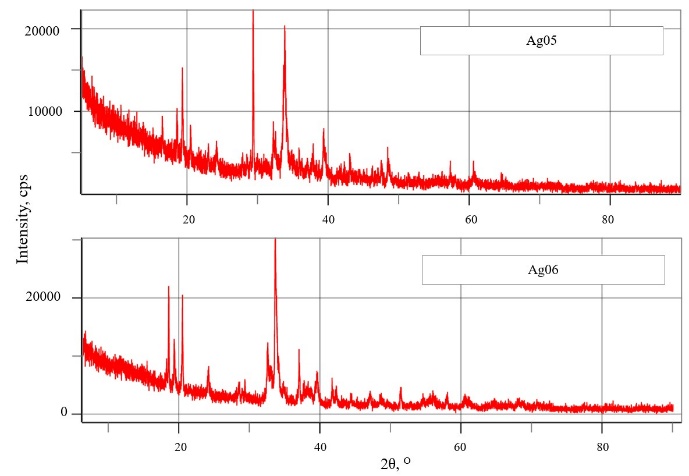


**Supplementary Figure 2: XRD pattern analysis of AgNPs synthesized. Ag03** M10 IsoEt is AgNPs synthesis by microwave assisted synthesis with 10 mM AgNO_3_ mixed with *I. galbana* hexane extract. **Ag04** M10 ChaEt is AgNPs synthesis by microwave-assisted synthesis with 10 mM AgNO_3_ mixed with *C. calcitrans* hexane extract. **Ag05** 1 mM AgNPs is AgNPs synthesized by the conventional method with 1 mM AgNO_3_ mixed with 0.1 M NaOH. **Ag06** 10 mM AgNPs is AgNPs synthesized by the conventional method with 10 mM AgNO_3_ mixed with 0.1 M NaOH.

Supplementary Figure 3


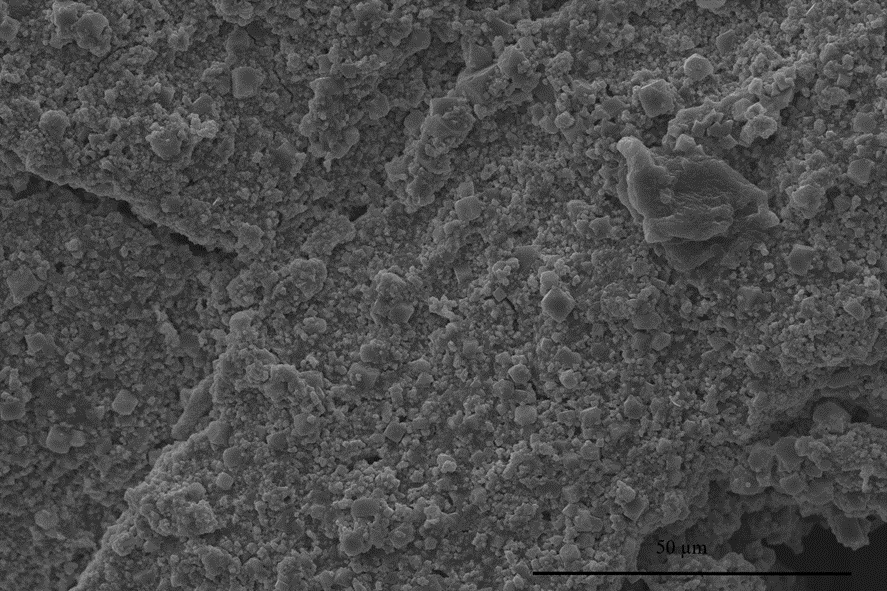
 **
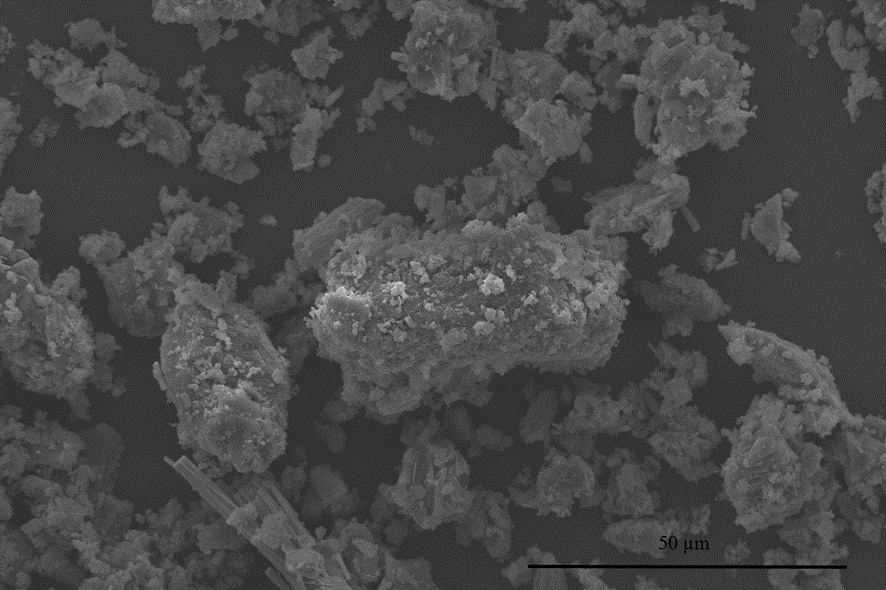
**

B

A


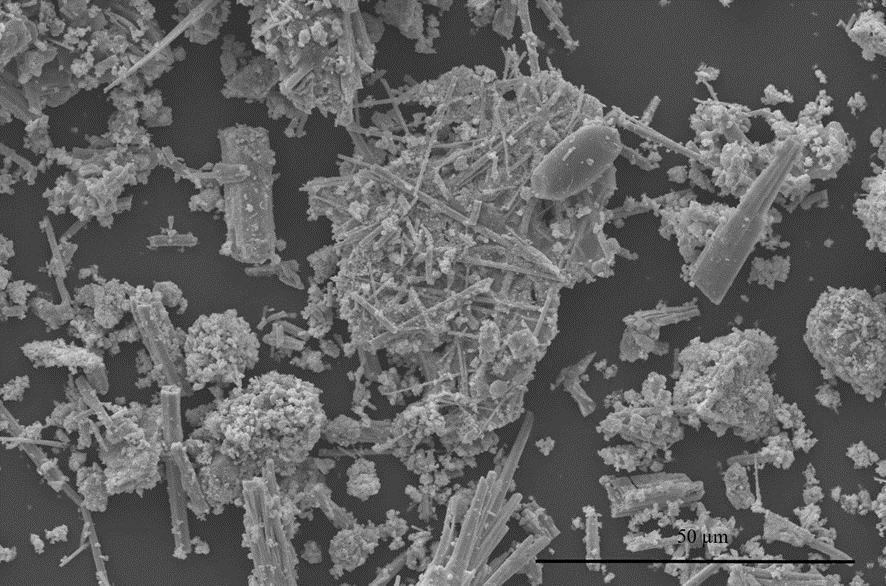


C

**Supplementary Figure 3:** **SEM image of AgNPs M10 IsoEt. A** M10 ChaEt is AgNPs synthesis by microwave-assisted synthesis with 10 mM AgNO_3_ mixed with *C. calcitrans* ethanolic extract. **B** 1 mM AgNPs is AgNPs synthesized by the conventional method with 1 mM AgNO_3_ mixed with 0.1 M NaOH. **C** 10 mM AgNPs is AgNPs synthesized by the conventional method with 10 mM AgNO_3_ mixed with 0.1 M NaOH. All images are displayed at a scale of 50 µm.

Supplementary Figure 4


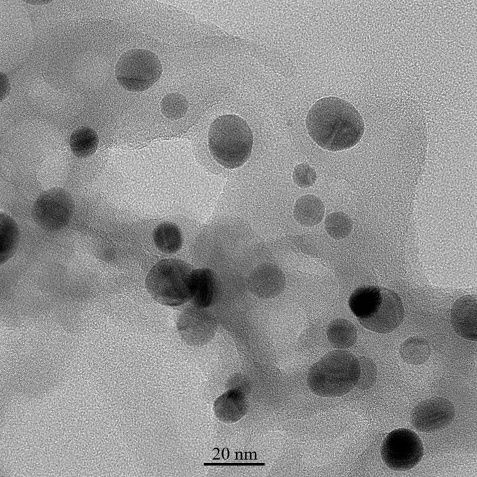

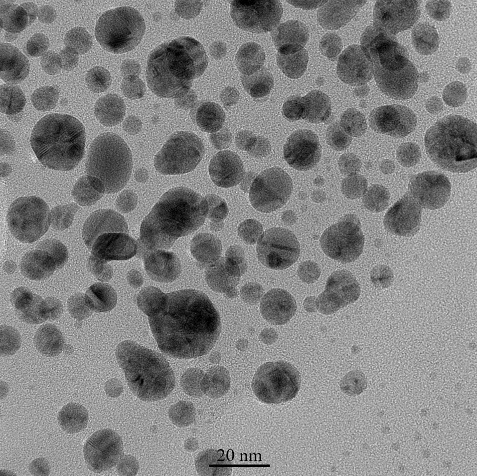


B

A

**
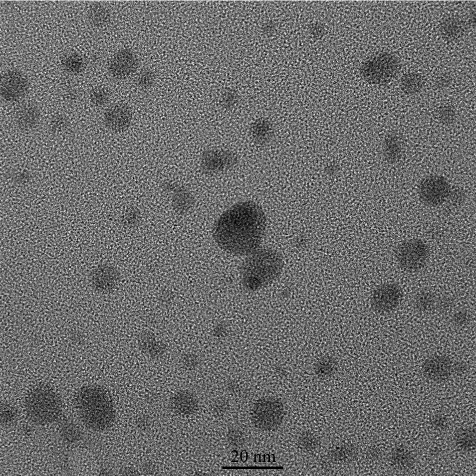

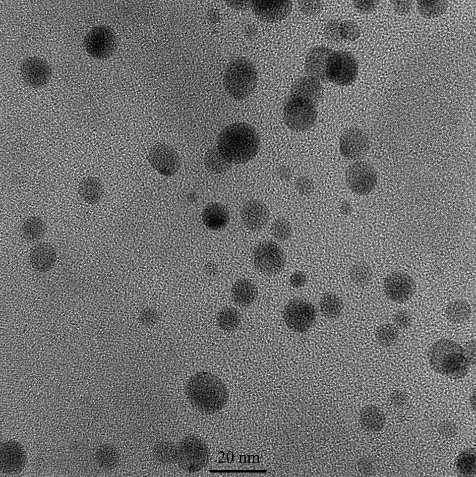
**

D

C

**Supplementary Figure 4: TEM image of AgNPs synthesized. A** 1 mM AgNPs exhibited particle sizes with minimal variation. **B** 10 mM AgNPs displayed a significant variation in particle sizes. **C** M10 ChaEt displayed a significant variation in particle sizes. **D** 10ChaEt displayed a significant variation in particle sizes. All images are displayed at a scale of 20 nm.
